# Radiofrequency thermocoagulation for V2/V3 idiopathic trigeminal neuralgia: effect of treatment temperatures on long-term clinical outcomes: A Cohort Study

**DOI:** 10.1097/MD.0000000000004019

**Published:** 2016-07-01

**Authors:** Peng Yao, Yi-yong Deng, Tao Hong, Zhi-bin Wang, Jia-ming Ma, Yong-qiang Zhu, Hong-xi Li, Yuan-yuan Ding, Shi-nong Pan

**Affiliations:** a Department of Pain Management, Shenjing Hospital of China Medical University, Shenyang; b Department of Pain Management, Siping Hospital of China Medical University (Siping Central Hospital of Jilin Province), Siping; c Department of Radiology, Shenjing Hospital of China Medical University, Shenyang, China.

**Keywords:** complications, pain relief, radiofrequency thermocoagulation, SF-36, trigeminal neuralgia

## Abstract

Radiofrequency thermocoagulation (RFT) is widely used to treat trigeminal neuralgia (TN); however, the optimal temperature at which RFT is most efficacious remains under much debate. Thus, the aim of the present study was to determine the lowest temperature at which morbidity could be minimized and patient outcomes maximized.

A multivariate analysis was used to study 1354 patients who underwent computed tomography (CT)-guided RFT for V2/V3 idiopathic trigeminal neuralgia (ITN) during from June 2006 to May 2015. RFT was carried out at 62, 65, and 68°C, while keeping all other RF parameters the same. This was a prospective cohort study, in which we assessed intra- and postoperative complications, pain relief, and long-term health-related quality of life (HRQoL).

The intraoperative and in-hospital complications of patients were mainly facial hematoma, mouth and external auditory meatus penetration, nausea, vomiting, dizziness, and headache, which were all treated symptomatically. In long-term follow-up, patients with pain relief (defined as no pain and no required drug intervention) at 62, 65, and 68°C accounted for 94.2%, 98.3%, and 98.8% (at discharge); 83.8%, 90.1%, and 91.4% (at 1 year); 66.7%, 80.5%, and 88.2% (at 3 years); 59.0%, 64.3%, and 77.2% (at 5 years); 48.7%, 57.8%, and 72.3% (at 7 years); 40.6%, 53.7%, and 60.3% (at 9 years), respectively. The number of patients with facial numbness, masticatory atonia, or corneal hypoesthesia was increased with the elevation of temperature, but these complications were all mild. No blindness, deafness, intracranial hemorrhage, or death as a result of the surgical intervention occurred in any patients. SF-36 scores showed highest HRQoL in the group treated at 68°C, followed by the 65 and 62°C groups, respectively.

Our results demonstrate that 68°C is a good choice for RFT of V2/V3 ITN. The alternative option is 65 or 62°C for RFT to minimize the occurrence of complications including facial numbness, yet which often yields a higher recurrence rate.

## 1 Introduction

Trigeminal neuralgia (TN) is the most common facial neuralgia, with a high morbidity and an incidence of 12.6 to 28.9/100,000 every year.^[1–3]^ TN can produce a fierce pain characterized by repeated, paroxysmal, severe neuralgia in a unilateral or bilateral facial distribution along regions of trigeminal nerve. The sequelae of the condition can influence activities of daily living such as speaking, eating, and tooth brushing. Although initial treatment for TN is generally pharmacological, when treatment fails, minimally invasive surgery is the major therapy for TN. Such surgical interventions include microvascular decompression,^[4]^ gamma knife radiosurgery,^[5]^ percutaneous balloon compression,^[6]^ radiofrequency thermocoagulation (RFT),^[7,8]^ pulsed radiofrequency (PRF),^[9,10]^ and intragasserian injection of substances such as Botox,^[11]^ phenol glycerite,^[6,12]^ and anhydrous alcohol.^[13]^

Of the aforementioned surgical interventions, many favor RFT due to the ability to perform this in a minimally invasive, highly efficient manner, with little mortality and morbidity, while at the same time achieve satisfactory functional outcomes. Short-term pain relief rates for patients treated with RFT was reported to be 90% to 100% in some studies,^[14–21]^ although the specific factors which produce the most successful outcomes are difficult to identify. For example, in review of these prior works, temperatures differed widely from study to study (60–95°C). Moreover, in some instances there was no mention of temperature differences for treatment of V1 (ophthalmic division) versus V2/V3 (maxillary division/mandibular division) TN, or alternatively no mention of temperature and RF parameters at all.^[14]^ It is well known that complications may result from RFT, and that serious complications such as facial palsy, blindness, deafness, and even death may result with high temperatures. RFT parameters in these studies differed greatly; an abnormal sensation of pain was reproduced with 50 Hz current stimulation at 0.1 to0.5 V/millisecond, but 100 Hz, 500 microseconds, and 1.0 mA were also used.^[21]^ Across the board, the diagnostic criteria for TN patients vary, and in 1 study idiopathic trigeminal neuralgia (ITN) was not distinguished from secondary trigeminal neuralgia (STN).^[20]^ Other studies have small sample sizes (as few as 5)^[22]^ and only a subset of these studies investigated short-term postoperative complications. Therefore, it is difficult to determine the efficacy of a single technique for treatment of TN, as the outcomes are highly variable and the clinical significance of the reported results is unknown.

Following the principle of “safety first” is very important, especially in China where the doctor–patient relationship is tense. Some studies report that RFT at 60 to 70°C and RFT at a higher temperature consistently resulted in pain relief, while causing fewer complications in 60 to 70°C.^[23,24]^ Although there is a high recurrence rate, secondary surgery is feasible after RFT, a technically matured mini-invasive surgery which can be completed within 30 to 40 minutes, is performed at a low temperature. Therefore, low-temperature RFT is necessary and feasible. The goals of the present study were to identify the optimal temperature for achieving efficacious treatment of ITN that would both optimize functional outcome and minimize complications.

## 2 Materials and methods

### 2.1 Patient population

This was a randomized, controlled, double-blinded, prospective cohort study in V2/V3 ITN patients. These patients underwent RFT at 62, 65, or 68°C, with a unified set of RFT parameters and were then observed for intraoperative and in-hospital adverse reactions, pain relief, TN recurrence, postoperative complications, and long-term health-related quality of life (HRQoL). This study was designed to provide clinical guidance.

All patients diagnosed with TN in the Department of Pain Management of Shengjing Hospital of China Medical University and the Department of Pain Management of Siping Hospital of China Medical University (Siping Central Hospital, Jilin Province) and planned to undergo subsequent treatment with RFT from June 2006 to May 2015 were evaluated and followed long term. This study was approved by the Ethics Committee of Shengjing Hospital of China Medical University. All patients provided informed consent.

The diagnoses of all patients were established following the diagnostic criteria for ITN or STN in the International Classification of Headache Disorders: 2nd edition (HIS-II) (2004).^[25]^ All patients were 28 years of age or older, had a preoperative pain score greater than 6 (VAS), suffered no facial numbness and received drug treatment for TN. RFT was recommended for patients whose pain was not controlled after the standard analgesic treatment of high-dose carbamazepine, gabapentin, or pregabalin continuously for over 3 months, and/or who experienced intolerable adverse effects, such as nausea, vomiting, dizziness, blurred vision, drowsiness, limb edema, and impaired liver function.

The following patients were excluded: patients with STN, including postherpetic TN, intracranial occupying lesions (including benign and malignant tumors) and multiple sclerosis; patients who previously underwent surgical treatment, including microvascular decompression, RFT, gamma knife radiosurgery, balloon compression, or other invasive interventions; patients with oromaxillofacial diseases including tumors; patients with concomitant hemorrhagic diseases; patients with severe cardiopulmonary insufficiency; patients with severe mental dysfunction; patients with V1 TN or complicated TN of V1, V2 and/or V3 were also excluded because primary therapy for V1 TN involves low temperature, peripheral RFT, or PRF.

A total of 1354 patients routinely underwent preoperative trigeminal and cranial magnetic resonance imaging (MRI) to exclude intracranial occupying lesions (e.g., hemangioma, cysts, benign and malignant tumors, etc.) and multiple sclerosis that might induce STN. Patients with vascular compression adjacent to the root and gasserian ganglion of trigeminal nerve were also included into the study.

At 30 minutes prior to surgery, reptilase 1 KU was injected intramuscularly, and ceftriaxone sodium 1 g (Shanghai Roche Pharmaceuticals Co. ltd., China) was infused intravenously. Immediately before operation, Innovar 2 mL(fentanyl 0.05 mg + droperidol 2.5 mg,Yichang Humanwell harmaceuticals Co. ltd., China) was slowly injected intravenously, and 2 mL was added during operation as necessary.

### 2.2 Surgical procedure

Gasserian ganglion RFT was performed by puncturing the foramen ovale using the Hartel anterior approach, followed by infiltration anesthesia with 0.5% lidocaine. Thereafter, a 22G RFT cannula (10 cm, with a 5 mm active tip) was introduced gently and slowly along the puncturing pathway to reach the periphery of the foramen ovale, and then the puncturing direction was adjusted by computed tomography (CT) scan to enter the foramen ovale. After no blood and cerebrospinal fluid (CSF) return was observed, the RF instrument was connected to induce division paresthesia correspondingly at 50 Hz, 1 millisecond, and 0.1 to 0.2 V. Local anesthesia was achieved with the injection of 0.2 mL of 1.5% lidocaine and RFT was performed 2 minutes later.

The patients were randomized to undergo RFT at different temperatures according to the random number table. RFT temperature was increased gradually from 50°C to the preset temperature (Group A: 62°C, Group B: 65°C, Group C: 68°C) and a lesion was then made for a total of 180 seconds. During RFT, the presence of eyelash reflex and facial numbness were repeatedly assessed. At the end of RFT, if pain was still present in the innervation zone of the trigeminal nerve, the location of the needle tip was further adjusted (2–5 mm puncturing or withdrawing), and then RFT at the corresponding temperature was performed after retesting. After surgery, patients were ordered to take 24 hours absolute bed rest (including 6 hours prostrated bed rest without pillow), with vital signs monitored for 6 hours; reptilase and antibiotics were administrated routinely 1 to 2 times. All patients were observed for 3 days postoperatively, and those with pain relief and no serious adverse reactions were discharged. Any patient who experienced complications and/or pain remained in the hospital for continuous observation and treatment.

### 2.3 Outcomes assessment

Pain relief, in-hospital complications (including facial hematoma, headache, nausea, vomiting, external auditory meatus bleeding, facial numbness, and pain at the puncture site), and treatment measures were recorded for each patient. In addition, those patients requiring propofol anesthesia due to surgical pain intolerance were noted.

At the time of hospital discharge, the patients’ level of pain relief was evaluated with the Barrow Neurological Institute (BNI) pain score, which incorporates both patient-rated pain as well as the degree of dependence on medication for pain control.^[26]^ The BNI scoring is as follows: I (excellent), the pain disappeared completely, requiring no drugs; II, the pain was mild, requiring no drugs; III, the pain was moderate, requiring drugs for complete control; IV, the pain was moderate, requiring drugs but not completely controlled; V, the pain was severe or not relieved. BNI scores I–III indicated satisfactory pain relief, while BNI scores IV–V indicated poor pain relief. All patients were followed up once per month in the first 6 months and once every 3 months thereafter and BNI was obtained at these follow-up visits.

Postoperative facial numbness was evaluated with the BNI facial numbness score, which incorporates the presence and degree of residual numbness as well as an indicator of the degree of bother to the patient. There were 4 levels of BIN facial numbness score: I, no numbness; II, mild numbness, causing no impact; III, moderate numbness, somewhat bothersome; IV, severe numbness, very bothersome.

The numbers of patients enrolled, lost to follow-up, and completing follow-up were recorded. The follow-up investigator was blinded to the patients’ surgical grouping. Follow-up was performed outpatient or by telephone, some patients could not pay a visit to the outpatient or communicate by telephone because of poor physical condition or hearing problems, performed family visit.

We assessed for postoperative complications including corneal hypoesthesia (corneal reflex test: when the patient stared at one side, a piece of cotton was used to gently touch the lateral-inferior side of the homolateral cornea in a lateral-to-medial direction and avoiding the patient saw it, so as to induce the contraction of bilateral musculus orbicularis oculi), masticatory atonia, and decreased hearing (hearing test). All complications were recorded in detail.

### 2.4 HRQoL evaluation

Postoperative physical and mental status were assessed with the Short Form-36 (SF-36).^[27]^ The physical evaluation included 4 parts: bodily pain (BP), role/physical (RP), physical functioning (PF), and general health (GH). The mental evaluation also included 4 parts: social functioning (SF), vitality/energy (VT), mental health (MH), and role/emotional (RE). The Physical Component Summary (PCS) and Mental Component Summary (MCS), which are the subtotals for physical and mental evaluations, were determined.^[28,29]^ The scores for single items in each part were summed to give a final score (a full score of 100). The greater scores indicated better patient status.

### 2.5 Statistical analysis

The quantitative data of normal distribution were analyzed with variance analysis of complete random design and then Baetlett test, and those of nonnormal distribution were analyzed with Kruskal–Wallis test. The categorical data were analyzed with chi-square test. The postoperative follow-up data of patients were analyzed with Kaplan–Meier survival curves and then log rank test, Breslow test, and Tarone–Ware test. The censored point was defined as loss of follow-up, TN recurrence, death, and the last visit. The patients who underwent RFT and injection treatment again were not included into the study repeatedly, and their follow-up was closed. The relations between age, sex, pre-RFT drug dosage, division of TN or intraoperative complications and postoperative pain relief were analyzed by Cox regression. All data were analyzed using IBM-SPSS 19.0 statistical software (Chicago, IL). *P* < 0.05 (2 sides, 95% confidence intervals) suggested that a difference was statistically significant.

## 3 Results

A total of 1354 patients meeting the inclusion criteria were included into the study. The general patient characteristics for each group are shown in Table 1. There were no statistically significant differences in age, sex, pre-RFT pain duration, follow-up duration, pre-RFT pain severity, drug (e.g., carbamazepine) dosage and SF-36 scores among various groups (*P* > 0.05). Any 1 patient with bilateral TN was regarded as 2 cases and underwent RFT at the same temperature for bilateral TN.

**Table 1:**
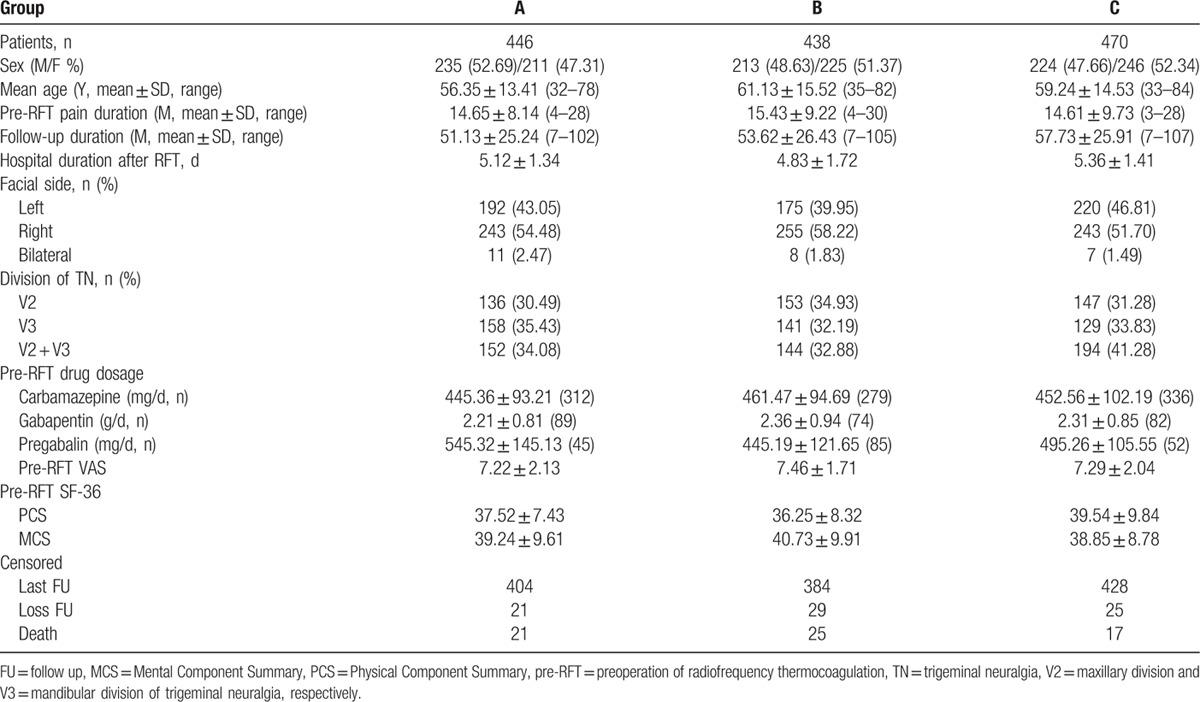
General patients characteristics.

### 3.1 Intraoperative complications and treatment measures

CT-guided RFT was successfully completed in all patients. The RFT time was ≤30 minutes in all cases. The intraoperative data and complications are shown in Table 2. A total of 52 (3.84%) patients experienced mouth penetration (mouth bleeding or bitter mouth after injection of local anesthetic drugs) by puncturing during local anesthesia or operation, including 20 patients, 17 patients, and 15 patients in Groups A, B, and C, respectively (*P* > 0.05). For those individuals the RFT cannula was replaced and the puncture was performed again. A total of 426 (31.6%) patients experienced facial hematoma during local anesthesia or intraoperative puncture (*P* > 0.05). In these instances, the surgical procedures were continued and a cold compress was applied postoperatively. A total of 310 (22.9%) patients experienced blood return after the puncture needle was delivered into foramen ovale, including 89, 114, and 107 patients in Groups A, B, and C, respectively (*P* > 0.05). The treatment in these instances consisted of puncture or withdrawal 3 to 5 mm after no blood return was observed, and even if the tested parameters were satisfactory, RFT was not performed at the initial position to prevent vascular wall injury and the resultant risk of postoperative intracranial hemorrhage. A total of 118 (8.72%) patients experienced CSF return, including 45, 38, and 35 patients in Groups A, B, and C, respectively (*P* > 0.05). When this occurred, RFT at the current position or the proper withdrawal of the puncture needle was performed based on the position of the needle tip with CT. No additional local anesthetic was injected. The temperature was slowly increased to the target temperature and the real-time eyelash reflex and forehead tests were conducted. If the patients could not tolerate the surgical pain, intravenous propofol 2 mg/kg was given as an anesthetic. In all, a total of 88 (6.50%) patients experienced a radiating pain of the external auditory meatus during intraoperative puncture. The puncture pathway was adjusted to prevent serious injury caused by a violent, rapid puncture, or RFT at a wrong position. When eyelash hypoesthesia, forehead numbness, or corneal hypoesthesia occurred during operation, RFT was aborted and the patients were asked and inspected about the pain area. If pain was still present or the patients could not locate the pain, the puncture needle was withdrawn 2 to 3 mm, the patients were reassessed with the previously described special tests, and then RFT was performed again. In Group C, 59 (12.55%) patients experienced eyelash hypoesthesia, 45 (9.57%) patients experienced forehead numbness and 37 (7.87%) patients experienced corneal hypoesthesia (*P* < 0.05) when compared with Group A. A total of 73 (5.39%) patients experienced bradycardia (heart rate (HR) < 60 bpm) or tachycardia (HR > 100 bpm) and 12 patients were treated symptomatically during operation; there were no differences among various groups. During the operation, 2, 2, and 3 patients in the 3 groups, respectively, experienced conscious ear discomfort, decreased hearing, or a blowing sensation. Of the number of patients requiring propofol anesthesia, there were no statistically significant differences between groups.

**Table 2:**
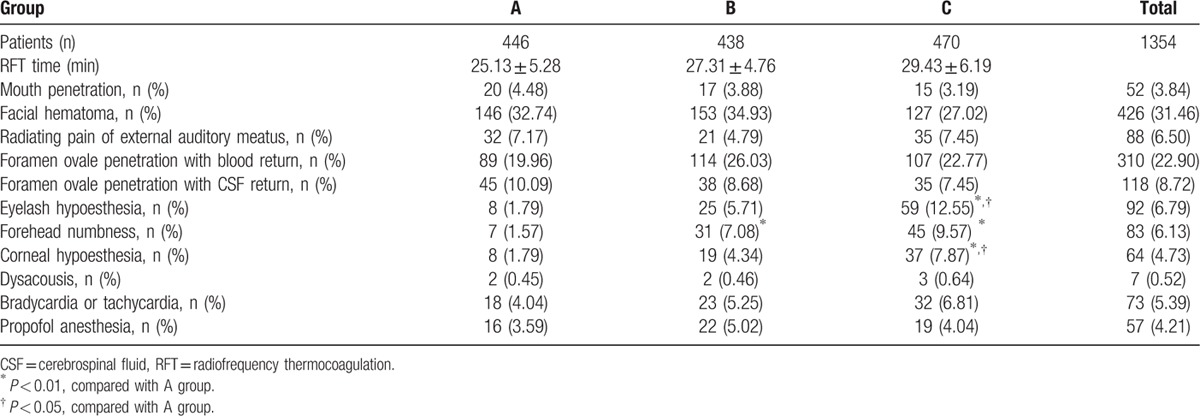
Intraoperative data and complications.

### 3.2 In-hospital complications and treatment measures

As shown in Table 3, headache, nausea, and vomiting occurring on postoperative day 1 were treated symptomatically with antiemetics. Severe headache was managed by a single intramuscular injection of nonsteroidal antiinflammatory drugs. All patients were closely observed and underwent CT scan as necessary, especially when there was suspicion for the probability of intracranial hemorrhage. All of the above symptoms recovered within 2 days. After operation, hyperalgesia in the innervation zone of trigeminal nerve occurred in 36, 41, and 55 patients within the 3 groups, respectively; this adverse reaction disappeared at postoperative days 3 to 4. Dizziness and headache after getting up indicated the patients had symptoms of intracranial hypotension (*P* > 0.05). These individuals were treated with 1500 mL/d of fluid replacement and bed rest. These symptoms were resolved after a period of 7 days. External auditory meatus bleeding was observed in 4, 5, and 3 patients within each of the 3 groups, respectively. Tympanic membrane perforation and decreased hearing as determined by the otologist occurred in four patients; all recovered at 1 month after symptomatic treatment. All other patients with decreased hearing recovered by the time of hospital discharge. After RFT, 15 patients in Group A, 13 patients in Group B, and 15 patients in Group C reported no pain relief with BNI scores IV–V and these individuals directly underwent secondary RFT during hospitalization. Facial numbness occurred in 8, 43, and 102 patients within 3 groups, respectively (*P* < 0.05). At discharge, facial numbness disappeared in 6 patients within Group A. In Groups B and C, facial numbness persisted to different degrees in 22 and 43 patients, masticatory atonia was found in 3 and 12 patients, and corneal hypoesthesia was noted in 2 and 14 patients, respectively. These complications were continuously assessed in subsequent follow-up. None of the patients developed fever. Despite the fact that some patients experienced complications, all were satisfied with RFT surgery.

**Table 3:**
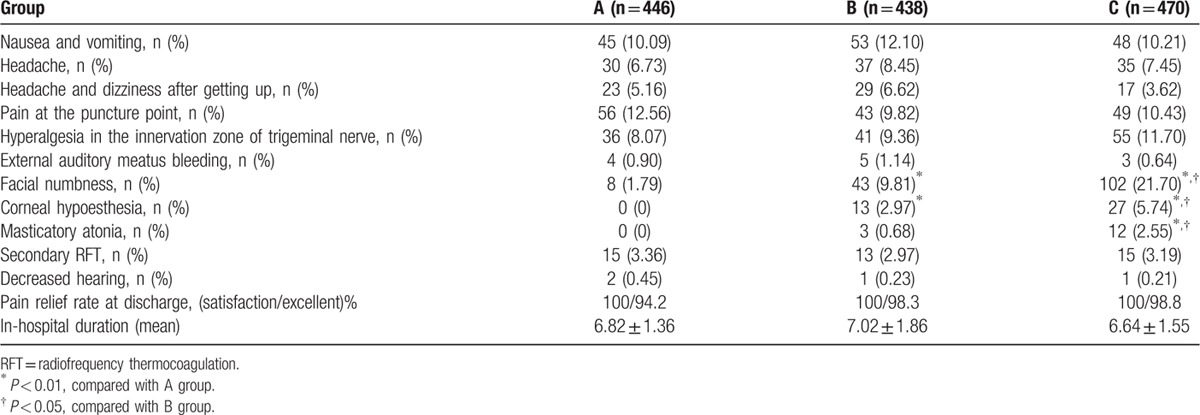
Short-term complications (in-hospital duration).

### 3.3 Long-term postoperative follow-up

During follow-up, 63 patients died (non-RFT causes) and 75 patients were lost to follow-up (*P* > 0.05, among 3 groups); these patients were regarded as censored cases. The survival time was defined as the time from discharge to the last visit. The remaining patients (Group A: 404 (90.58%), Group B: 384 (87.7%), Group C: 428 (91.6%) all completed follow-up). TN recurrence (BNI scores IV–V) was observed in 122 (27.35%), 91 (20.78%), and 59 (12.55%) patients within the 3 groups, respectively. A total of 95 patients underwent secondary RFT or blockage or other treatments, including 45 patients in Group A, 27 patients in Group B, and 23 patients in Group C. The follow-up deadline was the last visit before retreatment. The follow-up duration was 51.13 ± 25.24 (7–102) months in Group A, 53.62 ± 26.43 (7–105) months in Group B, and 57.73 ± 25.91 (7–107) months in Group C, respectively.

### 3.4 Postoperative pain relief

**Figure 1:**
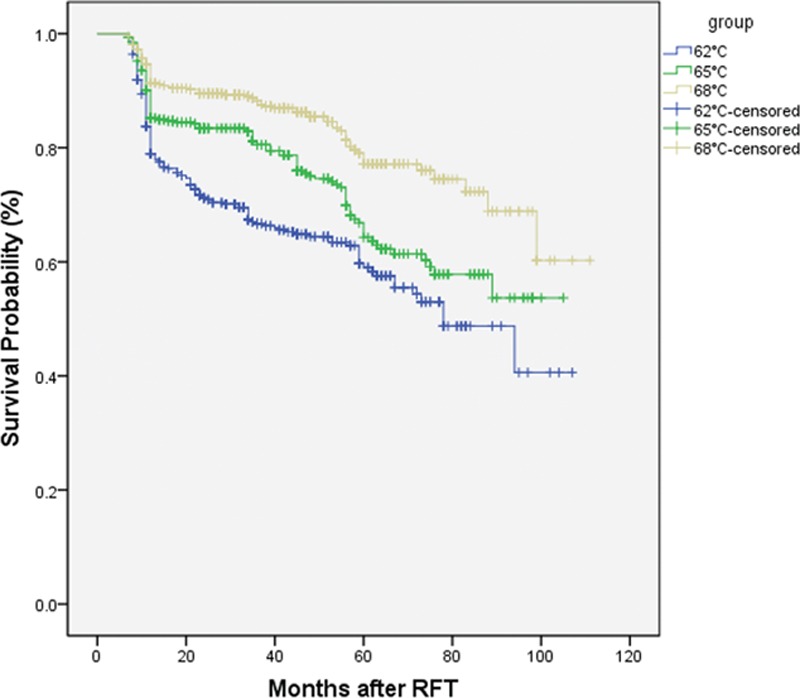
Kaplan–Meier curve indicate the outcomes of ITN patients without any pain (BNI I) after radiofrequency thermocoagulation (RFT).

**Figure 2:**
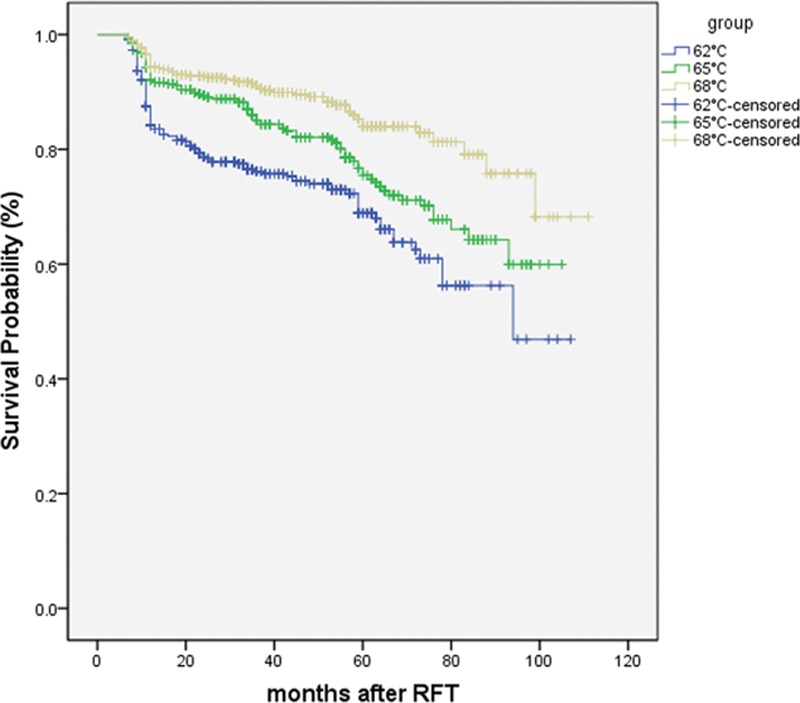
Kaplan–Meier curve indicate the outcomes of ITN patients (BNI I–III) after radiofrequency thermocoagulation (RFT).

The degree to which postoperative pain relief was achieved is shown in Figs. 1 and 2. Among the 3 groups, the percentage of patients with BNI scores I–III (satisfactory) at discharge was 100%, with the highest percentage of BNI score I (excellent) in Group C. The overall percentage of individuals with BNI scores I–III and BNI score of I declined with time, with a general trend of the highest treatment temperature (Group C) having the highest pain relief scores, and the lowest treatment temperature (Group A) having the lowest pain relief scores. This trend was consistent over the 9-year follow-up window. In Groups B and C, the patients with BNI score I accounted for >90% at 1 year and >80% at 3 years. In all groups, postoperative pain relief was not associated with the age, sex, division of TN, pre-RFT pain duration, pre-RFT analgesic dosage, and in-hospital duration of patients. At 9 years, the percentage of patients absolutely without pain was >50% in Groups B and C but only 40.6% in Group A.

### 3.5 Postoperative complications

Morbidity associated with facial numbness is demonstrated in Table 4. A total of 15 patients in Group C and 1 patient in Group B experienced moderate facial numbness (BNI score III), which resolved gradually over time (at 9.36 ± 6.17 months). At discharge, a total of 16 patients (Group A: 0, Group B: 2, Group C: 14) experienced corneal hypoesthesia, which resolved within half a year (at 3.63 ± 1.92 (2–6) months). The incidence rate of corneal hypoesthesia in Group C was higher than that in Group A or B. There were 0, 3, and 12 patients with masticatory atonia in Groups A, B, and C, respectively. Three patients in Group B and 10 patients in Group C recovered within half a year, and 2 patients in Group C recovered at 13 and 18 months. In this study, none of them experienced diplopia, blindness, deafness, or death as a result of RFT.

**Table 4:**
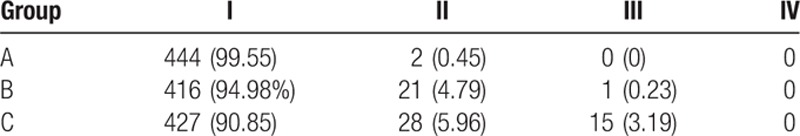
Facial numbness complications.

### 3.6 SF-36 HRQoL score

As shown in Table 5, PCS, MCS, and QOL scores were low in all 3 treatment groups prior to RFT. After RFT, PCS, and MCS were both significantly increased (72.3–73.8 and 74.3–76.1); the comparison before and after RFT was statistically significant (*P* < 0.01). In all groups, PCS and MCS gradually decreased over time, and patient satisfaction also declined. At 3 to 9 years after RFT, the comparisons among 3 groups showed statistically significant differences (*P* < 0.05).

**Table 5:**
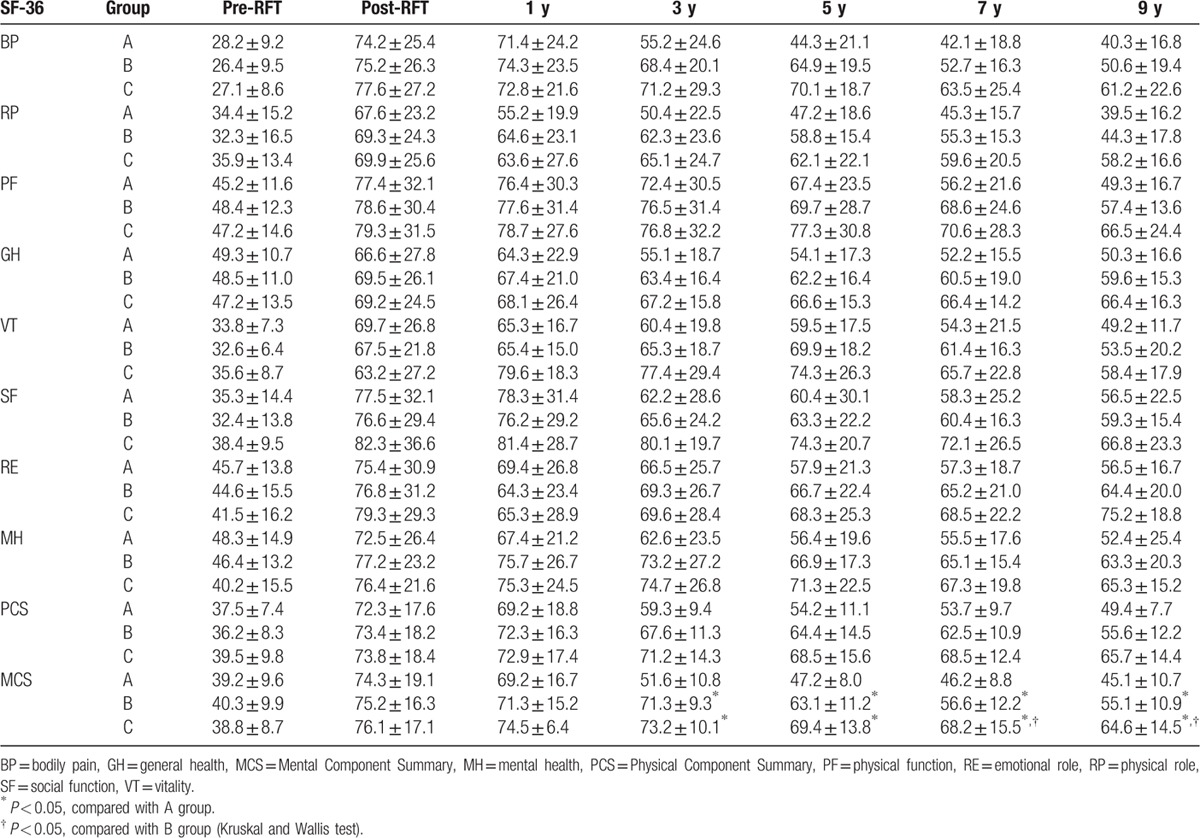
Comparison of SF-36 scores among the groups.

## 4 Discussion

This study was aimed to treat ITN by RFT at a lower temperature (62–68°C) which was relative to 70 to 95°C selected for RFT in some previous study reports.^[15,19–22,30,31]^ Postoperatively, we observed the efficacy and safety of RFT at 62 to 68°C in achieving pain relief during long-term follow-up to determine the most appropriate temperature at which RFT can be delivered in clinical practice. Our study results demonstrated that RFT at 65 to 68°C may be reasonable treatment temperatures for ITN, as treatment at these temperatures achieved satisfactory pain relief and had a lower incidence rate of complications. Although complications such as facial numbness and masticatory atonia were present in these 2 treatment groups, such complications were mild and resolved in a short time. No serious complications occurred as a result of RFT (e.g., blindness, deafness, death, etc.).

We selected temperatures of 62, 65, and 68°C for RFT based on several considerations: First, in our previous clinical practice, several patients experienced complications such as severe facial numbness, masticatory atonia, and salivation after RFT at 70 to 75°C, which seriously affected their HRQoL and even led to medical lawsuits. In searching the available literature, we found that a significant number of patients experienced serious complications (some of which were irreversible) after RFT at ≥70°C.^[8,20,30–32]^ Meanwhile, RFT at <70°C achieved acceptable pain relief but had fewer complications,^[23,24]^ which is confirmed by our study results. A study reported that in trans-foramen ovale RFT, the failure rate of surgical puncture was 4%^[33]^ while no failure of CT-guided surgical procedures happened in this study.

In the present study, satisfactory outcomes were observed after RFT at 62, 65, and 68°C, and the patient satisfaction with respect to pain relief (BNI I–III) was 100% at discharge, suggesting that short-term efficacy is determined by intraoperative technique. The percentage of patients absolutely without pain and requiring no analgesics (BNI I) was 94.2%, 98.3%, and 98.8% in 3 groups, which is consistent with the immediate pain relief rate obtained at a high temperature in the previous studies.^[16–18]^ Postoperatively, the general trend noted was that more patients experienced TN recurrence or the severity of pain was increased over time. For example, patients with initial scores of BNI I eventually had scores of BNI II–III or TN recurrence. There were fewer patients with BNI I in the 62°C group when compared with those in the 65 or 68°C groups. The number of patients with BNI I in the 65 and 68°C groups is higher than that in the studies of Haridas et al^[34–36]^ and consistent with that in the studies of Hart et al.^[37–40]^

In the present study, 15, 13, and 15 patients in 3 groups achieved no pain relief having BNI scores of IV–V in the immediate postoperative period. These patients were underwent secondary RFT and were then followed-up as per our protocol. Their inclusion into the study was reasonable and met the clinical treatment principles, as no pain relief was caused by the failure of assessment in the first RFT but not due to RFT technique itself.

A satisfactory analgesic effect was obtained at a low temperature, perhaps associated with the following aspects: Strict inclusion criteria were formulated, and HIS-II diagnostic criteria were referenced to include ITN and exclude STN. In some previous study reports,^[7,20,24,41]^ ITN was not strictly distinguished from STN, including STN caused by herpes zoster, multiple sclerosis, intracranial occupying lesions, postoperative recurrence, and other factors. Some study results also revealed that RFT had a poor pain relief effect for STN.^[14,34]^ There was a variation among RFT parameters selected. RFT parameters were not uniform in various reports,^[20,30,31]^ and some intraoperative parameters were not defined in many studies. The smaller stimulation parameters indicated that the tip of the RFT puncture needle was closer to the target position. In this study, 0.1 to 0.2 V parameters were selected for confirmation, and RFT was successfully completed under the precise location by CT guidance in all patients, though the surgically technical requirements were increased. V1 of TN was excluded in this study. The specificity of V1 requires treatment with RFT at a low temperature, or peripheral RFT or PRF to prevent V1 injury and subsequent serious complications such as corneal hypoesthesia, keratitis, and even blindness. As a result, RFT for V1 could not be performed at the same temperature as that for V2 and V3. We therefore, only investigated V2/V3 TN in the present study. However, there is a possibility that V1 TN might be explored in other study. It is also important to note that patients with V1 TN were included for RFT in the previous studies, which might influence the observation results. There was a small sample size in 1 study,^[22]^ which might cause a bias of statistical results and the failure to objectively evaluate the surgical technique.

Our study results demonstrated that long-term efficacy of pain relief was better in the 65 and 68°C groups than in the 62°C group, though there was a statistical difference between 65°C group and 68°C group. Fewer patients noted excellent pain relief in the 65°C group when compared with the 68°C group; however, pain relief was still maintained at a level commensurate with that mentioned in some previous reports. The long-term pain relief rate in the 62°C group was slightly lower than that in the previous follow-up reports of RFT, indicating that 62°C is too low to guarantee the long-term efficacy of pain relief. However, this temperature is suitable for those young patients who wish to experience no complications. From the perspective of achieving efficacy in pain relief, RFT at 65 to 68°C is preferable for treating ITN.

Long-term follow-up showed that the facial numbness and masticatory atonia were the major complications. In the present study, 2 (0.45%), 21 (4.79%), and 28 (5.96%) patients in the 62, 65, and 68°C groups, respectively, experienced mild facial numbness (BNI II). Moderate facial numbness (BNI III) was noted in 1 (0.23%) and 15 (3.19%) patients in the 65 and 68°C groups, respectively. Despite no statistical difference, as temperature was increased, more patients developed facial numbness and the severity of facial numbness was higher, which suggests that the temperature shall be carefully controlled for RFT. The percentage of patients with facial numbness in this study is far lower than the figures in some previous study reports, which ranged from 34.8% to 100%.^[8,20,30,38,41]^ Our study demonstrated that no patients experienced severe facial numbness, and those that did experience some degree of numbness recovered completely in a short time (i.e., 6.3 ± 5.6 (3–18) months). There were 3 (0.68%) patients in 65°C group and 12 (2.55%) patients in 68°C group who suffered from mild masticatory atonia; none of the patients in 62°C group experienced masticatory atonia. The incidence rates of masticatory atonia in various groups are all lower than the figures in the previous reports (8%^[8]^ and 46.7%^[21]^), and the recovery time of patients with masticatory atonia was 9.3 ± 4.6 (6–16) months. The patients with corneal hypoesthesia accounted for 2.97% in the 65°C group and 5.74% in the 68°C group, which indicates that the probability of corneal hypoesthesia is increased at a higher temperature. Corneal hypoesthesia might be a complication caused by the short distance between the RFT needle tip and V1 or by a high treatment temperature during RFT. Although strict intraoperative monitoring was performed, corneal hypoesthesia still occurred in some elderly patients who had a slow response and difficulties in communication. All patients with corneal hypoesthesia recovered within half a year (i.e., 3.6 ± 1.9 (2–6) months), which is consistent with prior reports in the literature.^[16,32,42]^ However, in the present study, the incidence of corneal hypoesthesia (2.97%/65°C, 5.74%/68°C) is lower than that reported in prior studies (18%^[43]^ and 19.4%–31.4%^[14]^).

Complications of RFT may include facial hematoma, headache, dizziness, or nausea and vomiting induced by intracranial hypotension and meningeal irritation. Moreover, postoperative pain at the puncture site and other complications can influence the postoperative evaluation of patients, though most patients recover rapidly within several days after operation. Meanwhile, the drugs (e.g., carbamazepine) used by the patients would be gradually decreased and discontinued over several days. Therefore, in order to isolate the treatment effect of RFT and remove the potential confounding effect of postoperative complications and/or pharmacologic therapy, follow-up of patients was initiated at the time of discharge. During RFT, 52 (3.84%) patients experienced mouth penetration, 12 patients experienced external auditory meatus penetration with bleeding, and 4 patients experienced tympanic membrane perforation with transient decreased hearing or blowing-like tinnitus (hearing recovered and tympanic membrane healed at 1 month). These findings suggested that decreased hearing was caused by tympanic membrane perforation, as opposed to auditory nerve injury. The 2 aforementioned intraoperative complications were caused by intraoperative maloperation, which is a defect of real-time observation in CT-guided surgical procedures. Therefore, the principle of gentleness and slowness must be followed throughout the puncture process, so that adjustments are made in a timely manner according to the actual situation when the patients have relevant reactions. Violent, rapid puncture, or RFT at a wrong position must be avoided to prevent serious injuries.

Patient HRQoL was evaluated with the SF-36 in this study. Many studies have proven that the SF-36 is a generally recognized good choice for the evaluation of patients HRQoL.^[27–29,44]^ Lam et al^[28]^ carefully observed the HRQoL of Chinese patients. The follow-up results in this study showed that the post-RFT HRQoL of ITN patients was well evaluated with SF-36. Before RFT, the patients suffered a fierce pain and had low PCS and MCS; after RFT, the pain was relieved and SF-36 scores were markedly increased in the postoperative window in all groups. The SF-36 scores in the 65 and 68°C groups were significantly greater than those in 62°C group, and overall patient satisfaction was improved in all groups.

In this study, the patients were conscious during RFT (i.e., under local anesthesia) to readily facilitate communication, exchange, and evaluation of their status in real-time in an effort to prevent serious complications. An economic burden was also one consideration, about RMB 1500 yuan was saved for each patient by the use of local anesthesia. The feasibility of such a technique has been proven by some studies on the intragasserian injection of phenol glycerite, anhydrous alcohol, Adriamycin or local anesthetic drugs combined with steroids.^[11–13,38,45]^ In review of literature in the field, a recent study by Lin et al^[38]^ reported patient outcomes when RFT was carried out under local anesthesia. So, the choice of local anesthesia was reasonable and feasible. But there are some shortcomings and deficiencies in terms of evaluation of pain relief scores. During RFT under local anesthesia, some patients experience severe pain, which influences the postoperative patient satisfaction evaluations. Following the use of local anesthesia, any or all 3 divisions of TN are numb, influencing the effect evaluation during RFT. In the present study, no pain relief (BNI IV–V) was observed in 43 patients after initial RFT, and the pain was noted to disappear in all these patients after repeat RFT. These results are related to poor position in order to avoid blood vessels and the reevaluation failure after local anesthesia during RFT. In addition to meningeal irritation signs in RFT, the incidence of postoperative nausea and vomiting is probably associated with the use of local anesthetic. At the same time, eyelash hypoesthesia, forehead numbness, or corneal hypoesthesia occurred during operation perhaps associated with a difference in temperature or due to the use of local anesthetic drugs, yet which was uncertain during RFT.

However, general anesthesia also has its own disadvantages. In this instance, while recovering consciousness and being able to communicate, patients are still under superficial anesthesia state and experience residual analgesic and sedative effects, which will affect the intermediate intraoperative evaluation and thus greatly prolong the operation time.

Recurrence after treatment is an important consideration in the treatment of TN. Reportedly, the efficacy of RFT for TN is mainly dependent on the reduction of demyelinated nerve fiber synaptic transmission,^[46]^ rather than the thorough destruction of these nerve fibers by increasing the temperature. It is also important to note, however, that RFT position is known to play a role in reducing TN recurrence.^[47]^ Additionally, Luo et al pointed out that satisfactory pain relief of TN could be achieved by PRF at 42°C by increasing RF field intensity and output voltage.^[10,48]^ Taken together, the aforementioned studies suggest that increasing temperature is only one way in which postoperative TN recurrence may be reduced. During follow-up in the present study, TN recurrence was observed in 122 (27.35%), 91 (20.78%), and 59 (12.55%) patients within the 62, 65, and 68°C groups, respectively. These present findings are consistent with previous study results, which reports that the recurrence occurred in 14.5% to 51.5% of the patients but this variability was due to differences in temperature (≥70°C), parameter settings, etc.^[8,30,49–51]^ Prior studies reported that a temperature ≤80°C could selectively block action potentials of Aδ and C nerve fibers, and that at higher temperatures Aα and Aβ nerve fibers action potentials are blocked.^[31,38,52]^ These considerations are often accounted for in temperature selection for clinical RFT and some observational studies. However, there is a need to further evaluate clinical RFT at such temperatures. Yang et al^[23]^ achieved satisfactory pain relief of TN by RFT at 60°C, proving the utility of RFT at a low temperature, which is supported by the results of the present study. Given that the trigeminal nerve is a mixed motor nerve, high temperature RFT may destroy neurons or axons, thereby reducing overall nerve function.

Our study has some limitations. First, there are no uniform specifications in the previous study reports of TN treatment^[53,54]^; however, these parameters such as experimental design, case selection, temperature, anesthetic, voltage, current should be standardized to the maximal extent. Our study excluded V1 TN, it is known that our study outcomes do not completely represent those for all cases of treating ITN with RFT. Additionally, the same RFT parameters rather than several different RFT parameters were used, thus making it difficult to perform related comparisons between temperature with different parameters. Lastly, we did not compare the effects of general anesthesia vs local anesthesia on RFT, nor did we conduct a comparative observation by selecting higher temperatures. Each of the aforementioned limitations are areas that may be further explored in subsequent studies.

## 5 Conclusion

RFT is effective and safe for ITN treatment; however, appropriate temperature selection is an issue worthy of wide concerns, because it is directly related to postoperative pain relief outcomes, as well as intra- and postoperative complications. RFT at a high temperature may improve pain relief and decrease recurrence, but this is at the risk of serious complications including facial numbness, masticatory atonia, blindness, deafness, and even death. In addition to temperature, RFT efficacy is closely associated with the surgical procedures, RFT parameters, and electrode position. Our study results demonstrate that RFT at low temperatures can achieve satisfactory pain relief with fewer and milder postoperative complications than when carried out at higher temperatures. Therefore, RFT at 68°C is recommended for treating V2/V3 ITN, and RFT at 62 to 65°C is optional for patients who wish to minimize the complications such as facial numbness.
